# Reconstitution of internal target volumes by combining four-dimensional computed tomography and a modified slow computed tomography scan in stereotactic body radiotherapy planning for lung cancer

**DOI:** 10.1186/1748-717X-9-106

**Published:** 2014-05-02

**Authors:** Seong Soon Jang, Gil Ja Huh, Suk Young Park, Po Song Yang, Hae Nam Chung, Jae Hyuk Seo, Ji Chan Park, Young Jun Yang, Eun Youn Cho

**Affiliations:** 1 Department of Radiation Oncology, Daejeon St. Mary’s Hospital, College of Medicine, The Catholic University of Korea, 222 Banpo-daero, Seocho-gu, Seoul 137-701, South Korea; 2 Department of Internal Medicine, Daejeon St. Mary’s Hospital, College of Medicine, The Catholic University of Korea, Seoul, South Korea; 3 Department of Radiology, Daejeon St. Mary’s Hospital, College of Medicine, The Catholic University of Korea, Seoul, South Korea; 4 Department of Radiology, Daejeon St. Mary’s Hospital, Daejeon, South Korea; 5 Department of Radiation Oncology, Bucheon St. Mary’s Hospital, Bucheon, South Korea; 6 Department of Radiation Oncology, Daejeon St. Mary’s Hospital, Daejeon, South Korea

**Keywords:** 4D CT, Slow CT, Internal target volume, Lung cancer, Stereotactic body radiotherapy

## Abstract

**Background:**

To evaluate the volumetric and geometric differences in the ITVs generated by four-dimensional (4D) computed tomography (CT), a modified slow CT scan, and a combination of these CT methods in lung cancer patients treated with stereotactic body radiotherapy (SBRT).

**Methods:**

Both 4D CT and modified slow CT using a multi-slice CT scanner were performed for SBRT planning in 14 patients with 15 pulmonary targets. Volumetric and geometric analyses were performed for (1) ITVall, generated by combining the gross tumor volumes (GTVs) from all 8 phases of the 4D CT; (2) ITV2, generated by combining the GTVs from 2 extreme phases of the 4D CT; (3) ITVslow, derived from the GTV on the modified slow CT scan; (4) ITVall+slow, generated by combining ITVall and ITVslow; and (5) ITV2+slow, generated by combining ITV2 and ITVslow. Three SBRT plans were performed using 3 ITVs to assess the dosimetric effects on normal lung caused by the various target volumes.

**Results:**

ITVall (11.8 ± 8.3 cm^3^) was significantly smaller than ITVall+slow (12.5 ± 8.9 cm^3^), with mean values of 5.8% for the percentage volume difference, and a mean of 7.5% of ITVslow was not encompassed in ITVall. The geometric coverages of ITV2 and ITVslow for ITVall were 84.7 ± 6.6% and 76.2 ± 9.3%, respectively, but the coverage for ITVall increased to 90.9 ± 5.9% by using the composite of these two ITVs. There were statistically significant increases in the lung-dose parameters of the plans based on ITVall+slow compared to the plans based on ITVall or ITV2+slow. However, the magnitudes of these differences were relatively small, with a value of less than 3% in all dosimetric parameters.

**Conclusions:**

Due to its ability to provides additional motion information, the combination of 4D CT and a modified slow CT scan in SBRT planning for lung cancer can be used to reduce possible errors in true target delineation caused by breathing pattern variations.

## Background

Stereotactic body radiotherapy (SBRT) has been widely adopted for medically inoperable early-stage non-small cell lung cancer (NSCLC) and metastatic lung cancer with high local control rate of > 80% and acceptable toxicity in most studies [1]. In addition, for centrally located tumors in the lung, SBRT can be feasible with a less aggressive dose regimen and the exclusion of critical structures from the high-dose region. Despite promising results in medically inoperable patients, the role of SBRT in treating operable NSCLC remains to be defined by ongoing randomized trials. This approach involves the delivery of an ablative dose to the target using highly conformal and hypofractionated radiation over a short time course. In radiotherapy for lung cancer, large uncertainties exist in target delineation and localization because of respiration-induced tumor motion. These uncertainties are particularly influential in the SBRT technique, which uses high doses in small fractions for a small target volume.

The magnitude of respiration-induced tumor motion in the lung can be greater than 2 cm depending on the tumor location and the patient. In fact, this motion exhibits a clear patient-specific aspect, as it is difficult to estimate the range of motion before actual measurements [2-4]. The International Commission on Radiation Units and Measurements (ICRU) Report 62 introduced the concept of an internal target volume (ITV), which consists of the clinical target volume (CTV) with the addition of an internal margin to account for tumor motion [5]. In addition, the American Association of Physicists in Medicine (AAPM) has produced a report on methods for reducing the impact of respiratory motion, including motion-encompassing methods [6]. Individualized ITVs that encompass the entire range of respiratory tumor motion can be effectively constructed using the following three computed tomography (CT) acquisition methods: slow CT, inhalation and exhalation breath-hold CT, and four-dimensional (4D) CT scans. Slow CT involves scanning using a slow gantry rotation speed to ideally capture the full range of tumor motion within each slice, while producing blurred images of moving objects [6]. Recently, an adaptation of the 4D CT technique allowed for the acquisition of 3D CT images at multiple phases of the respiratory cycle. This capability has proven very useful in the planning of radiotherapy by considering the respiratory motions of the tumor and organs [7]. The use of data from a 4D CT scan, which is the gold standard for ITV delineation, is also strongly recommended for SBRT planning in the case of lung cancer [8]. There are several methods for using 4D CT data to generate individualized ITVs [4,9]. One time-consuming method, which may be the most accurate method of acquiring ITVs using 4D CT, is the delineation of a composite volume that encompasses the gross tumor volumes (GTVs) in all respiratory phase bins. A useful tool for reducing the clinical workload involved in contouring on all 4D CT phases is to use the maximum intensity projection (MIP) dataset or only the 2 extreme phases of the 4D CT scan.

However, these methods based on a single 4D CT scan, which is sampled from just a few breathing cycles, might underestimate the full extent of the true tumor motion during treatment due to possible variations in the patient’s breathing pattern [4,10]. Despite breath coaching to ensure a regular breathing pattern or motion monitoring at the time of treatment with image guidance techniques, differences in breathing patterns between the 4D CT planning and the treatment delivery can lead to geometric errors. In addition to techniques for regular breathing and image guidance, studies have compared the differences in target volumes obtained using different CT scan techniques for patients with lung cancer [11-15]. The addition of motion information from an additional CT scan method might serve to reduce the risk caused by breathing pattern variations. As an attempt to develop a more accurate ITV delineation method in SBRT planning for lung cancer, we focused on the combined use of 4D CT and a modified slow CT scan. The present study was undertaken to evaluate the volumetric and geometric differences in the ITVs generated by 4D CT, a modified slow CT, and the combination of these CT methods in lung cancer patients treated with SBRT. In addition, the dosimetric consequences for lung organs at risk (OAR) in SBRT planning using various ITV definitions were compared for these patients.

## Methods

### Patient characteristics

With the approval (DIRB-00102_1-002) of the institutional review board of Daejeon St. Mary's Hospital, 15 tumors in 14 patients treated with SBRT for early-stage NSCLC (13 tumors) or pulmonary metastases (2 tumors) at our institution between December 2009 and June 2013 were included in this retrospective study. The median age of the patients was 66 years (range: 55–86 years), and the sample included 13 males and 1 female. The tumor location was the upper lobe for 9 tumors, the middle lobe for 1 tumor, and the lower lobe for 5 tumors. Of these 15 tumors, 14 tumors were peripherally located and 1 tumor was centrally located.

### CT data acquisition and tumor motion analysis

Both 4D CT and modified slow CT techniques using a multi-slice CT scanner (SOMATOM Sensation 64; Siemens Medical Solutions, Erlangen, Germany) were performed for SBRT planning in all patients. Patients were advised to breathe freely and regularly. First, a single helical 4D CT scan including the whole lung was acquired with fixed acquisition parameters (pitch of 0.1, rotation time of 0.5 seconds, 120 kV, and 400 mA) and a commercially available motion-monitoring system (AZ-733 V; Anzai Medical, Tokyo, Japan). A pressure sensor (AZ-733 V) fixed in the upper abdominal region by means of an elastic belt generated the external respiratory signal. A lower signal amplitude (low pressure) corresponds to the exhalation phase, and a higher amplitude (high pressure) corresponds to the inhalation phase of the breathing cycle. Abdominal compression for the reduction of breathing motion was not applied for any patient. Using the Syngo software package (Siemens Medical Solutions), the projections were sorted retrospectively based on the respiratory signal, and the images were reconstructed into 8 respiratory phase bins equally distributed over the breathing cycle, with a slice thickness of 3.0 mm. Immediately following the 4D CT scan, a modified slow CT scan with the same scan range and slice thickness was performed with the same scanner, using the longest possible gantry rotation time, 1.0 s, and a reduced pitch factor of 0.5 [16]. The amplitude of the tumor motion was determined by measuring the tumor movement in the 8-phase 4D CT datasets using the InSpace 4D software package (Siemens Medical Solutions). The motion ranges at the tumor centroid in the superior-inferior (SI), anterior-posterior (AP), and left-right (LR) directions were measured in the transverse, sagittal, and coronal planes with a grid spacing of 1 mm for all 8 phase bins registered by this software.

### Target volume definitions

All CT datasets were transferred into a commercial treatment-planning system (Pinnacle^3^ version 8.0 m; Philips Medical Systems, Fitchburg, WI, USA), and thereafter, the 4D CT and modified slow CT images were superimposed using an automated algorithm of the Syntegra® software package (Philips Medical Systems). Match results were visually verified by reviewing the alignment of the spinal vertebrae. GTVs in each of the 8 phases of the 4D CT image were delineated using lung window setting by the same radiation oncologist and projected onto the modified slow CT image of the same slice. We used 5 approaches to define the ITVs: (1) combining the GTVs from all 8 phases of the 4D CT (ITVall); (2) combining the GTVs from 2 extreme phases (end-exhalation and end-inhalation) of the 4D CT (ITV2); (3) contouring the GTV on the modified slow CT scan (ITVslow); (4) combining ITVall and ITVslow (ITVall+slow); and (5) combining ITV2 and ITVslow (ITV2+slow) (Table 1).

**Table 1:** Summary of different ITVs and investigated parameters for volumetric and dosimetric changes

**ITV**	**Definition**
ITVall	Volume generated by combining the GTVs from all 8 phases of 4D CT
ITV2	Volume generated by combining the GTVs from 2 extreme phases of 4D CT
ITVslow	Volume derived from the GTV on a modified slow CT scan
ITVall+slow	Volume generated by combining ITVall and ITVslow
ITV2+slow	Volume generated by combining ITV2 and ITVslow
**Change**	**Parameter**
Volume	Absolute volume, PVD
Shape/Location	POV, Geometric coverage (Missing volume) for a specific ITV
Lung-dose	MLD, V5, V10, V20, V25, V30

*Abbreviations:ITV* internal target volume; *GTV* gross tumor volume; *4D CT* four-dimensional computed tomography; *PVD* percentage volume difference; *POV* percentage of overlap volume, defined as the ratio between the overlapping and encompassing volume; *MLD* mean lung dose; V5, V10, V20, V25, and V30, percentage volumes of both lungs minus the PTV receiving more than 5, 10, 20, 25, and 30 Gy.

### Volumetric and geometric analyses

Five target volumes obtained using different ITV definitions were measured for each of the 15 tumors, and the percentage volume difference (PVD), defined as |Va - Vb| / Va, between each pair of ITVs was also calculated. In addition to these volumetric differences, the following parameters were measured to compare the geometric differences caused by changes in target shape and location for each tumor: the percentage of overlap volume (POV), defined as the ratio between the overlapping and encompassing volume between each pair of ITVs; the geometric missing volume of ITVall for ITVslow; the geometric coverage of ITV2, ITVslow, and ITV2+slow for ITVall; and the geometric coverage of ITVall and ITV2+slow for ITVall+slow. To evaluate the impact of the tumor parameters on the volumetric and geometric differences among the ITVs, the PVD and POV between each pair of ITVs were correlated with the tumor parameters, such as the mean GTV and motion range factors (SI, AP, and LR movement; 3D mobility; and overlap ratio between the 2 extreme bins). The volumes among ITVs were also compared in subgroups corresponding to the upper/middle lobe and the lower lobe, according to the tumor location.

### Dosimetric analysis

Three conformal SBRT plans for all 15 tumors were performed using 3 ITVs of ITVall, ITVall+slow, and ITV2+slow to assess the dosimetric effects on a normal lung resulting from the various target volumes. The planning target volumes (PTVs) were created by adding a uniform 5 mm margin to the ITVs. All plans used 10–14 coplanar and/or non-coplanar beams and were normalized such that at least 95% of the PTV received the prescription dose. The dose-fractionation schedules were 48 Gy in 4 fractions (13 tumors), 56 Gy in 4 fractions (1 tumor), and 50 Gy in 5 fractions (1 tumor). To provide a meaningful comparison, the beam energies, weights, and gantry angles were held fixed for each tumor. The dosimetric effects on a normal lung of SBRT planning using the 3 different ITVs were analyzed via lung-dose parameters such as the mean lung dose (MLD) and the percentage volumes of both lungs minus the PTV receiving specific doses of 5, 10, 20, 25, and 30 Gy (V5, V10, V20, V25, and V30), as estimated using dose-volume histograms.

### Statistical analysis

To compare the volumetric and geometric differences between each pair of ITVs and the lung-dose parameters for the three SBRT plans, we used a Wilcoxon signed-rank test for each tumor. The correlations between the volumetric and geometric differences and the tumor parameters for each tumor were evaluated using Spearman correlation analyses. Values of P < 0.05 were regarded as significant. All statistical analyses were performed using the SPSS software package (version 15.0; SPSS Inc., Chicago, IL, USA).

## Results

The tumor motions were the most extensive in the SI (6.2 ± 4.2 mm) direction, approximately 1.6 times greater than those in the AP (3.9 ± 2.2 mm) and LR (3.7 ± 2.6 mm) directions. The mean 3D mobility, which was calculated as (SI^2^ + AP^2^ + LR^2^)^1/2^, was found to be 8.3 ± 5.2 mm for all 15 tumors, and the tumors exhibited distinct differences in 3D mobility corresponding to their location in the lung. The 3D mobility for tumors in the upper/middle lobe (n = 10) was 5.1 ± 2.3 mm, and that for the lower lobe (n = 5) was 14.7 ± 2.4 mm (p < 0.01). The overlap ratios between the 2 extreme bins for tumors in the upper/middle lobe and the lower lobe were 0.65 ± 0.05 and 0.39 ± 0.11, respectively (p = 0.086).

Table 2 presents the measurements of mean GTV for the GTVs from all 8 phases and the 5 ITVs generated using different ITV definitions. On average, the volumes were obtained in the following order: ITVslow (9.7 ± 7.0 cm^3^) ≈ ITV2 (10.0 ± 7.5 cm^3^) < ITV2+slow (11.5 ± 8.3 cm^3^) ≈ ITVall (11.8 ± 8.3 cm^3^) < ITVall+slow (12.5 ± 8.9 cm^3^). Comparisons between each pair of ITVs exhibited statistical significance (p < 0.01), except between ITV2 and ITVslow (p = 0.820) and between ITVall and ITV2+slow (p = 0.140). Interestingly, there was a significant difference between ITVall and ITVall+slow (p = 0.001), and the PVD between them was 5.8 ± 3.5% (range: 1.6 – 13.5%). The PVD with respect to ITVall was decreased by a factor of approximately 3 for ITV2+slow (5.3 ± 4.8%) compared to the PVD between ITV2 and ITVall (15.3 ± 6.6%).

**Table 2:** Mean GTV and the 5 ITVs defined for all tumors

**Tumor no.**	**Mean GTV (cm**^**3**^**)**	**ITVall (cm**^**3**^**)**	**ITV2 (cm**^**3**^**)**	**ITVslow (cm**^**3**^**)**	**ITVall+slow (cm**^**3**^**)**	**ITV2+slow (cm**^**3**^**)**
1	4.9	7.9	6.9	5.1	8.1	7.4
2	11.5	13.8	11.9	12.4	14.2	13.1
3	12.4	23.4	20.6	21.6	25.5	24.4
4	5.2	7.4	6.1	5.8	7.5	6.7
5	1.9	2.8	2.3	2.8	3.2	3.1
6	8.3	14.0	9.1	9.9	15.1	11.5
7	7.6	13.6	11.7	11.9	14.4	13.6
8	2.3	4.0	3.7	3.0	4.1	3.9
9	4.3	8.7	7.0	7.7	10.1	8.9
10	4.7	6.1	5.5	5.8	6.7	6.4
11	9.4	12.3	11.0	10.9	12.9	12.3
12	3.5	6.9	5.5	5.3	7.3	6.2
13	1.3	1.8	1.5	1.4	1.8	1.7
14	16.0	24.0	20.3	15.9	25.6	23.6
15	24.7	30.1	27.5	25.4	31.5	29.5
Mean ± SD	7.9 ± 6.3	11.8 ± 8.3	10.0 ± 7.5	9.7 ± 7.0	12.5 ± 8.9	11.5 ± 8.3

**Figure 1:**
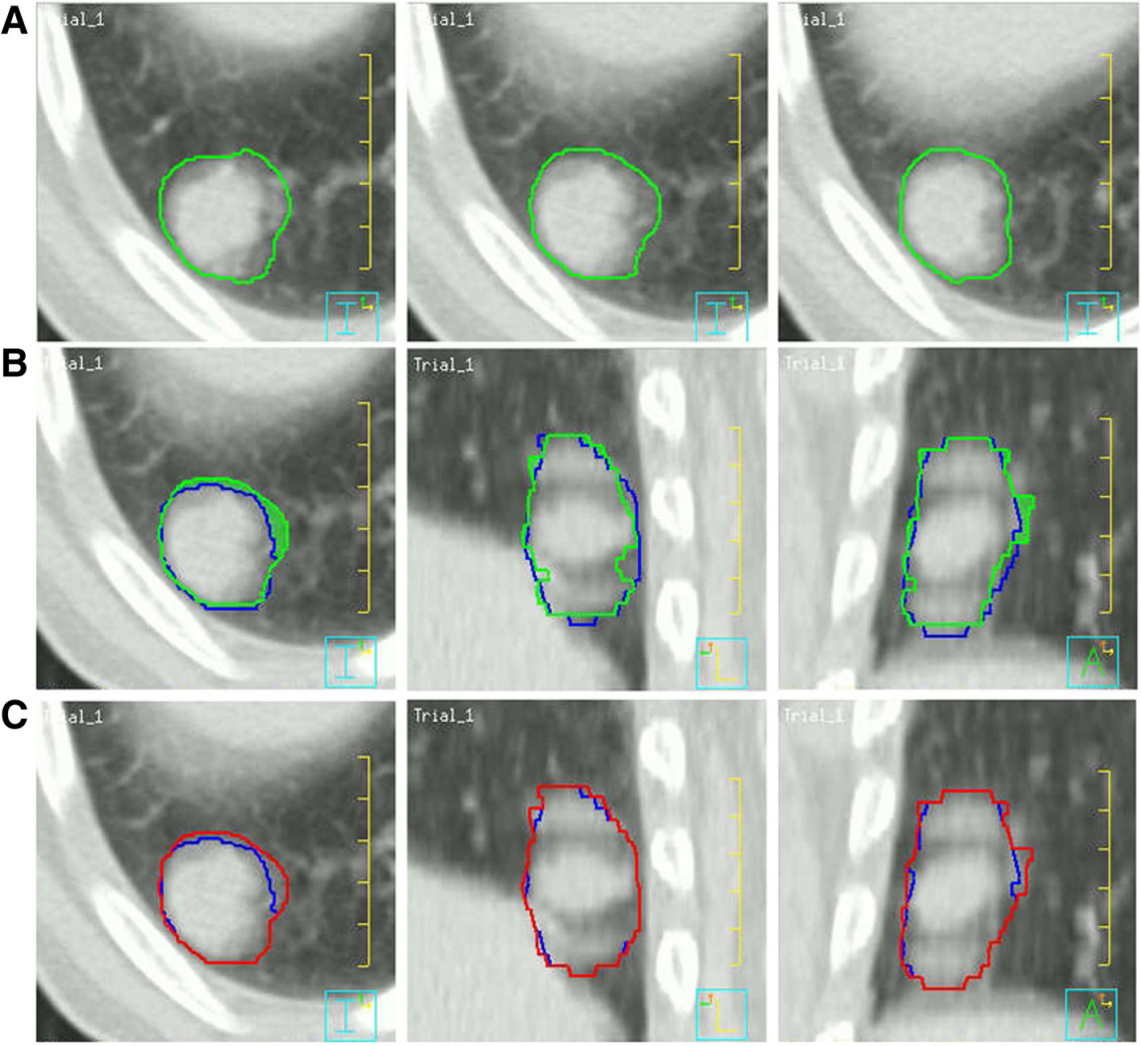
**Example of different ITVs in a lung cancer patient (tumor No. 3).** Transverse, sagittal, and coronal views of ITVslow (green), ITVall (blue), and ITVall+slow (red) are projected onto a modified slow CT scan. **A**. Delineation of ITVslow. **B**. Relationship between ITVall and ITVslow. The geometric regions of ITVslow that are missed by ITVall are displayed as green colored regions. **C**. Relationship between ITVall and ITVall+slow.

**Figure 2:**
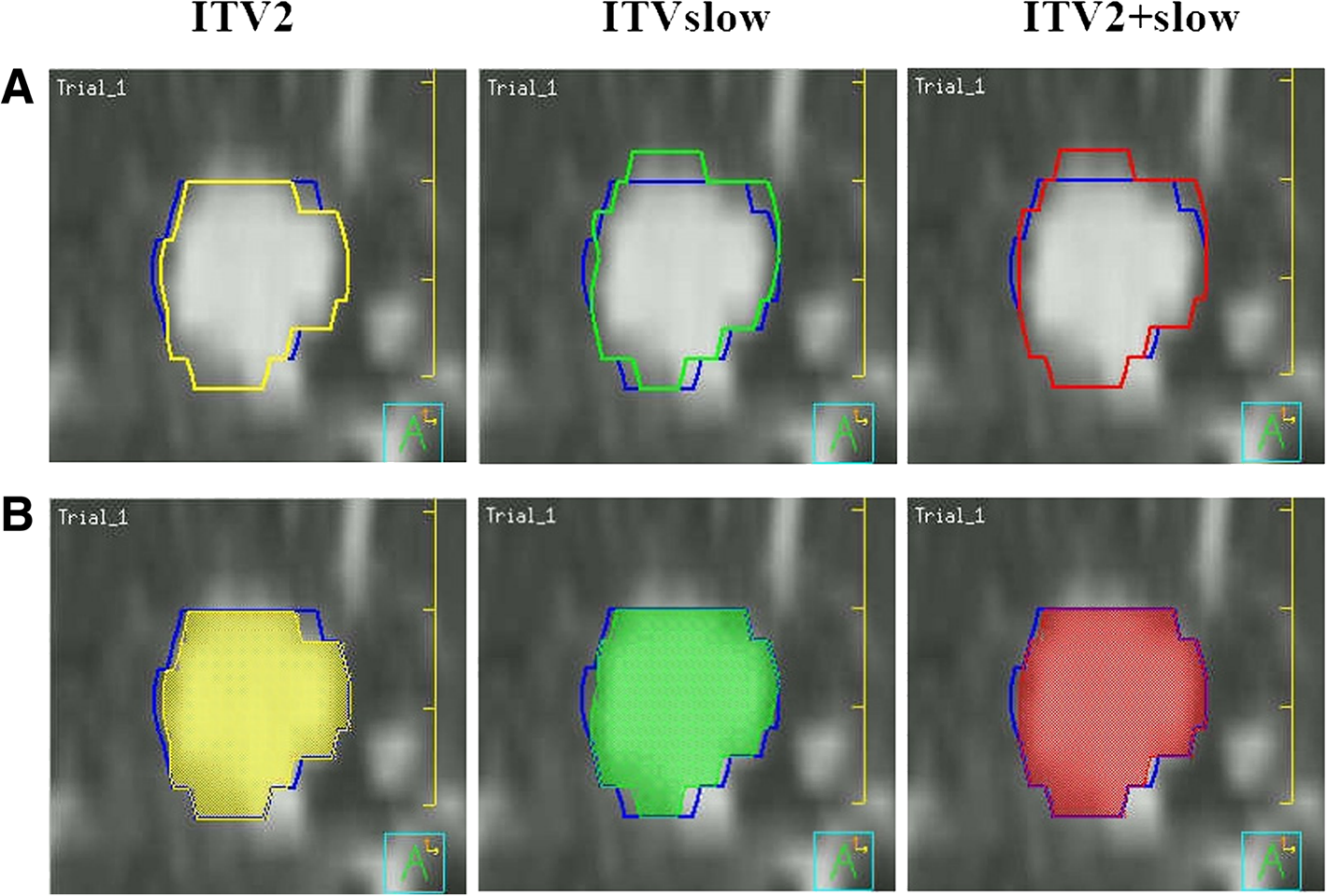
**Geometric coverages of 3 ITVs for ITVall in a lung cancer patient (tumor No. 10). A**. Relationship between ITVall (blue) and ITV2 (yellow), ITVslow (green), and ITV2+slow (red) in coronal views. **B**. Geometric coverages of ITV2, ITVslow, and ITV2+slow for ITVall.

In the geometric analyses among the ITVs, a mean of 7.5% (range: 2.0 – 17.6%) of ITVslow was not encompassed in ITVall. The geometric coverages of ITV2 (POV between ITV2 and ITVall) and ITVslow for ITVall were 84.7 ± 6.6% and 76.2 ± 9.3%, respectively. However, the coverage for ITVall increased to 90.9 ± 5.9% when using the composite (ITV2+slow) of these two ITVs (p < 0.01), and the difference in geometric coverage (POV) for ITVall+slow between ITVall (94.2 ± 3.5%) and ITV2+slow (91.5 ± 5.4%) was not significant (p = 0.140) (Figures 1 and 2, Table 3). No PVD or POV between any pair of ITVs exhibited a significant correlation with tumor parameters such as the mean GTV or motion range factors. In addition, the variations in the volumetric differences among the ITVs for the subgroups of the upper/middle and lower lobes were not statistically significant.

**Table 3:** Geometric coverage between ITVs based on different ITV definitions

**Tumor no.**	**Coverage (%) for ITVall**			**Coverage (%) for ITVall+slow**		**Coverage (%) for ITVslow**
	**ITV2**	**ITVslow**	**ITV2+slow**	**ITVall**	**ITV2+slow**	**ITVall**
1	86.6	61.7	90.2	97.5	90.4	96.0
2	85.7	87.6	92.0	97.8	92.2	97.5
3	88.1	83.6	95.4	91.9	95.7	90.5
4	83.4	77.5	88.9	98.4	89.0	98.0
5	80.2	86.2	95.0	88.4	95.5	86.9
6	65.3	62.7	74.2	92.8	76.1	89.0
7	86.3	81.5	93.6	94.1	94.0	92.9
8	91.9	71.6	95.6	97.3	95.7	96.4
9	79.9	72.9	86.4	86.5	88.2	82.4
10	89.8	85.9	95.9	91.8	96.2	90.6
11	90.1	84.0	95.2	95.0	95.4	94.1
12	80.4	70.2	83.3	93.8	84.4	91.4
13	87.2	78.6	93.0	97.9	93.0	97.5
14	84.6	59.7	91.8	93.6	92.3	89.8
15	91.1	79.6	93.4	95.5	93.7	94.5
Mean ± SD	84.7 ± 6.6	76.2 ± 9.3	90.9 ± 5.9	94.2 ± 3.5	91.5 ± 5.4	92.5 ± 4.4

Among the SBRT plans based on 3 different ITVs, there were statistically significant increases in the MLD, V5, V10, V20, V25, and V30 of the plans utilizing ITVall+slow due to an increase in the target volume compared to the plans employing ITVall or ITV2+slow. The V20 values for the plans that used ITVall+slow, ITVall, and ITV2+slow were 5.10 ± 1.90%, 4.97 ± 1.88%, and 5.00 ± 1.86%, respectively. However, the magnitudes of these differences among the plans were relatively small, with a value of less than 3% for all dosimetric parameters, and the differences in lung-dose parameters between the plans that used ITVall and ITV2+slow were not significant (Table 4).

**Table 4:** Differences in lung-dose parameters for SBRT plans based on 3 different ITVs

**Parameters**	**ITVall+slow**	**ITVall**	**ITV2+slow**	**ITVall+slow vs. ITVall**		**ITVall+slow vs. ITV2+slow**		**ITVall vs. ITV2+slow**	
	**(Mean ± SD)**	**(Mean ± SD)**	**(Mean ± SD)**	**Mean (range)**	***p*** *	**Mean (range)**	***p*** *	**Mean (range)**	***p*** *
MLD (cGy)	357.3 ± 89.7	351.6 ± 89.6	353.1 ± 88.1	5.8 (−1.2-33.9)	0.001	4.3 (0.1-13.5)	0.001	−1.5 (−33.8-12.3)	0.887
V5 (%)	17.05 ± 3.90	16.88 ± 3.88	16.94 ± 3.87	0.18 (−0.07-1.04)	0.011	0.11 (−0.01-0.47)	0.001	−0.06 (−1.04-0.54)	0.910
V10 (%)	11.77 ± 2.98	11.57 ± 2.94	11.67 ± 2.98	0.20 (−0.05-1.38)	0.008	0.10 (0.01-0.23)	0.001	−0.10 (−1.37-0.17)	0.820
V20 (%)	5.10 ± 1.90	4.97 ± 1.88	5.00 ± 1.86	0.14 (0.00-0.86)	0.001	0.10 (0.00-0.30)	0.001	−0.03 (−0.85-0.24)	0.776
V25 (%)	3.37 ± 1.37	3.28 ± 1.37	3.29 ± 1.31	0.09 (−0.01-0.55)	0.001	0.09 (0.00-0.37)	0.001	0.00 (−0.55-0.30)	0.496
V30 (%)	2.28 ± 0.98	2.22 ± 0.98	2.22 ± 0.93	0.06 (0.00-0.37)	0.001	0.06 (0.00-0.27)	0.001	0.00 (−0.38-0.21)	0.496

*Wilcoxon signed-rank test.

## Discussion

Respiration-induced tumor motion in the lung exhibits patient-specific patterns and thus is generally difficult to estimate. However, studies concerning the measurement of tumor motion in the lung using various methods, including 4D CT, commonly report that the largest motions primarily occur in the SI direction and the lower lobe [2,3,17]. We also observed greater motion in the SI direction and the lower lobe, with patient-specific aspects. In radiotherapy for lung cancer, this type of tumor motion suggests the need for an individualized margin that considers motions within the patient’s breathing cycle rather than the application of population-based margins; these individual approaches are considered to be especially important for the SBRT technique. Considering the magnitude of tumor mobility, motion-reducing methods such as gating and abdominal compression can be applied during SBRT for lung cancer. However, the most frequently used technique is the continuous delivery of static beams under free breathing. When patients breathe freely, the target volume must be adjusted to completely encompass the tumor in all phases of the respiratory cycle. However, the motion-encompassing ITV definitions based on 4D CT imaging might lead to the use of an unnecessarily large safety margin during treatment because these definitions do not consider the fact that the tumor spends unequal durations in different portions of its trajectory [4]. More importantly, although breath training, audio-visual biofeedback, or image-guidance techniques can be used to ensure regular breathing during CT acquisition and treatment, the single 4D CT scan that is commonly used in SBRT planning is a snapshot image corresponding to only a few breathing cycles at the time of CT acquisition. Therefore, ITV methods based on a single 4D CT scan might underestimate the full extent of the potential motion during treatment because they do not reflect the true tumor motion caused by breathing pattern variations during actual treatment [4,10]. Before 4D CT was widely adopted in radiotherapy planning for lung cancer, a slow CT technique with a scanning time of 4 s per slice was used as a method of accurately defining moving target volumes, based on the fact that the breathing period in patients with lung cancer was no longer than 4 s in most cases. In several studies, such slow CT scans have been shown to generate larger and more reproducible target volumes for lung tumors than fast CT scans, thereby indicating their greater ability to capture tumor movement [11,18,19]. However, movement during a long scanning time can lead to obscure images of the tumor and adjacent anatomic structures, and the loss of resolution caused by such motion blurring leads to larger observer errors in delineating the tumor and normal organs [6]. Using a low pitch factor, slow CT scanning can be effectively performed in a modern CT scanner, with the limitation of the gantry rotation time, which is intended for a fast scan speed [16]. Although our modified slow scan differs from the conventional slow CT scanning conducted at 4 s per slice, the data acquisition time for each slice increased from 0.36 s for our fast CT protocol in the thorax (gantry rotation time of 0.5 s and pitch of 1.4) to 2.0 s for this modified slow CT scan. This modified slow CT scan provides images with a more representative geometry for the entire respiratory cycle, as would occur during treatment, that can be used as reference images for dose calculation; this approach minimizes the image blurring observed for full slow CT scans and provides additional motion information that is complementary to the single 4D CT results. However, a limitation of our methods might be some exposure by additional CT scan. Regarding to the doses for these CT scans, the mean values of dose-length product (DLP) and effective dose for the 4D CT scan in all patients were 918.3 ± 111.5 mGy · cm and 12.9 ± 1.6 mSv, respectively, and additional doses for the modified slow CT scan were 163.5 ± 17.9 mGy · cm and 2.3 ± 0.3 mSv, respectively.

In this study, ITVslow was the smallest among all ITVs and was statistically different from all other ITVs except ITV2. Thus far, few studies have directly compared the target volumes determined using slow CT and 4D CT in the same patients. In a study in which both slow CT (rotation time of 4 s) and 4D CT were performed using a 4-slice CT scanner for SBRT of lung tumors [14], no significant difference in size was found between target volumes determined using the two CT techniques. However, the size of the target volume generated from slow CT was smaller than that generated from all phases of 4D CT by a mean value of 25%. Furthermore, based on the results of a group study investigating the difference between breathing periods of < 4 s and ≥ 4 s, the authors suggested that the motion patterns of tumors during CT scanning may be more important than the breathing period in differentiating these target volumes. In our study using a modified slow CT scan, the ratio of ITVslow/ITVall was 0.82 on average, and the PVD between these two ITVs exhibited a mean value of 17.5%, with a significant difference in the ITV sizes obtained with the two different CT techniques. This difference may be a result of the smaller target volume in this slow CT technique, as the tumor motions over the full respiratory cycle at each couch position could not be adequately covered because of the faster scan speed compared to the conventional slow CT speed of 4 s per slice. Another possible explanation is that the difference may result from a change in tumor motion caused by breathing pattern variations between the two CT techniques; the possibility of such an effect was suggested by Nakamura et al. [14]. In addition to this volumetric difference, our observation about the geometric coverage between ITVslow and ITVall is consistent with those reported by Nakamura et al. [14], who found that a mean of 8% of the target volume derived from slow CT was not encompassed in the target volume derived from all phases of 4D CT. Based on this geometric difference between the ITVs generated from 4D CT and modified slow CT, ITVall was significantly smaller than ITVall+slow. In a recent study, Ge et al. [15] compared volumetric differences among ITVs based on 4DCT, including a method that used a GTV contoured on a fast 3D CT image (GTV3D) in lung cancer patients treated with SBRT. In this research, the ITV generated by all 10 phases of 4DCT (ITV10phase) and the ITV generated by combining ITV10phase and GTV3D were 14.7 cc and 15.9 cc, respectively, indicating a significant volumetric difference. The authors also suggested that individualized ITV’s uncertainties could be minimized by combining target volumes generated from 4D CT and fast 3D CT. Currently, ITV methods using a single 4D CT scan, which is considered the gold standard for SBRT planning, might underestimate the true tumor motion during treatment due to possible variations in breathing patterns between the planning CT acquisition and treatment [4,10], but this inaccuracy in target delineation might be reduced by the addition of motion information from an additional CT technique.

The clinical workload involved in contouring on all 8–10 bins within the breathing cycle can be reduced by the tools using the MIP or only the 2 extreme phases of 4D CT [4,9]. However, the value of MIP scans is limited in cases where the tumor is adjacent to normal structures, such as the chest wall or mediastinum and MIP-based ITVs tend to underestimate the true tumor motion in comparison to ITVs that use all phases of the respiratory cycle [20,21]. When the magnitude of the motion is small compared to the tumor size, the range of motion can be effectively contoured using only the 2 extreme phases. However, in the case of small highly mobile tumors, intermediate tumor positions are required to generate a reliable ITV [4,9]. In our study on the use of 2 extreme phases, the geometric coverage of ITV2 for ITVall was 84.7%. Other studies concerning target volumes have employed only the 2 extreme phases of 4D CT. Ezhil et al. [21] reported results similar to ours in 17 patients with stage I lung tumor; they found that the ITVs based on the 2 extreme phases covered 83.9% of the ITVs generated from all 10 phases. Furthermore, Rietzel et al. [22] reported that 10 patients with stage I-III lung cancer exhibited a 93.7% overlap between the PTVs generated from only 2 extreme tumor positions compared to those generated from 10 respiratory phases. Our study also exhibited an increased value of 90.8% when comparing the overlap between 2 such PTVs after adding a uniform 5 mm setup margin to the 2 ITVs, and these volumetric and geometric differences with respect to ITVall were decreased by combining ITV2 and ITVslow, indicating that there is no significant volumetric difference between ITVall and ITV2+slow. Therefore, this composite ITV using 2 extreme phases and a modified slow scan may be useful for more accurate target delineation compared to the ITV method alone using 2 extreme phases. In particular, this composite method may be helpful for constructing more reliable ITVs for small and highly mobile tumors. Using correlation and subgroup analyses, we found that the volumetric and geometric differences among the ITVs exhibited consistent results for all tumors, regardless of tumor parameters such as the mean GTV, location, or motion range factors. This finding indicates that the respiratory tumor motions were sufficiently reflected by ITV2 and ITVslow, which may differ from the target volumes generated using fast CT or the end-exhalation phase. Indeed, the correlations for the PVD and POV between ITVall and ITVslow with respect to the 3D mobility were not significant.

Recent studies have suggested that the MLD and the percentage of total lung volume receiving a specific dose can serve as the main dosimetric predictors of symptomatic radiation pneumonitis after SBRT. For MLD ≤ 5 Gy and/or V20 ≤ 5-10%, the risk of grade ≥ 2 radiation pneumonitis found in most studies is only 10-15% [23-26]. In our results regarding dosimetry on a normal lung with respect to the variations in the derived ITVs, the values of lung-dose parameters were significantly increased in the plans based on ITVall+slow in comparison to the plans based on ITVall or ITV2+slow, but the magnitudes of these differences among plans were relatively small. Therefore, considering the possible error in true target delineation caused by breathing pattern variations and the small dosimetric difference in normal lung tissue caused by the increased target volume, the target volume definition obtained by combining 4D CT and a modified slow CT scan may be preferable to that obtained using a single 4D CT scan.

## Conclusions

We found that ITV delineation based solely on 4D CT and that based on 4D CT plus a modified slow CT scan were significantly different in volumetric and geometric comparisons. The combination of 4D CT and a modified slow CT scan in SBRT planning for lung cancer can be used to reduce possible errors in true target delineation caused by breathing pattern variations, as this approach provides additional motion information. ITV delineation using only the 2 extreme phases of 4D CT plus a modified slow CT scan may be appropriate as a strategy for reducing the clinical workload, as this method generates a more reliable ITV than techniques utilizing only the 2 extreme phases of 4D CT.

## Abbreviations

SBRT: Stereotactic body radiotherapy; NSCLC: Non-small cell lung cancer; ITV: Internal target volume; CTV: Clinical target volume; 4D CT: Four-dimensional computed tomography; GTV: Gross tumor volume; MIP: Maximum intensity projection; SI: Superior-inferior direction; AP: Anterior-posterior direction; LR: Left-right direction; ITVall: Volume generated by combining the GTVs from all 8 phases of 4D CT; ITV2: Volume generated by combining the GTVs from 2 extreme phases of 4D CT; ITVslow: Volume derived from the GTV on a modified slow CT scan; ITVall+slow: Volume generated by combining ITVall and ITVslow; ITV2+slow: Volume generated by combining ITV2 and ITVslow; PVD: Percentage volume difference; POV: Percentage of overlap volume, defined as the ratio between the overlapping and encompassing volume; PTV: Planning target volume; MLD: Mean lung dose; V5, V10, V20, V25, and V30: The percentage volumes of both lungs minus the PTV receiving more than 5, 10, 20, 25, and 30 Gy; SD: Standard deviation.

## Competing interests

The authors declare that they have no competing interests.

## Authors’ contributions

SSJ designed the study, did all the analysis, and wrote the draft of the manuscript. SSJ, GJH and SYP collected the data and performed the statistical analysis. PSY and HNC provided useful comments for the 4D CT and modified slow CT scan techniques. JHS and EYC were involved in treatment planning and dose measurements. JCP and YJY participated in the analysis of the data and helped with editing the manuscript. All authors read and approved the final manuscript.
